# Homeobox A7 increases cell proliferation by up-regulation of epidermal growth factor receptor expression in human granulosa cells

**DOI:** 10.1186/1477-7827-8-61

**Published:** 2010-06-14

**Authors:** Yu Zhang, Qing Huang, Jung-Chien Cheng, Yoshihiro Nishi, Toshihiko Yanase, He-Feng Huang, Peter CK Leung

**Affiliations:** 1 Department of Reproductive Endocrinology, Women's Hospital, School of Medicine, Zhejiang University, Hangzhou, 310006, China; 2 Department of Obstetrics and Gynaecology, Child and Family Research Institute, University of British Columbia, Vancouver, British Columbia, V6H 3V5, Canada; 3 Department of Medicine and Bioregulatory Science, Graduate School of Medical Sciences, Kyusyu University, Fukuoka, 812-8582, Japan; 4 Department of Endocrinology and Diabetes Mellitus, School of Medicine, Fukuoka University, Fukuoka, 814-0180, Japan

## Abstract

**Background:**

Homeobox (HOX) genes encode transcription factors, which regulate cell proliferation, differentiation, adhesion, and migration. The deregulation of HOX genes is frequently associated with human reproductive system disorders. However, knowledge regarding the role of HOX genes in human granulosa cells is limited.

**Methods:**

To determine the role of HOXA7 in the regulation and associated mechanisms of cell proliferation in human granulosa cells, HOXA7 and epidermal growth factor receptor (EGFR) expressions were examined in primary granulosa cells (hGCs), an immortalized human granulosa cell line, SVOG, and a granulosa tumor cell line, KGN, by real-time PCR and Western blotting. To manipulate the expression of HOXA7, the HOXA7 specific siRNA was used to knockdown HOXA7 in KGN. Conversely, HOXA7 was overexpressed in SVOG by transfection with the pcDNA3.1-HOAX7 vector. Cell proliferation was measured by the MTT assay.

**Results:**

Our results show that HOXA7 and EGFR were overexpressed in KGN cells compared to hGCs and SVOG cells. Knockdown of HOXA7 in KGN cells significantly decreased cell proliferation and EGFR expression. Overexpression of HOXA7 in SVOG cells significantly promoted cell growth and EGFR expression. Moreover, the EGF-induced KGN proliferation was abrogated, and the activation of downstream signaling was diminished when HOXA7 was knocked down. Overexpression of HOXA7 in SVOG cells had an opposite effect.

**Conclusions:**

Our present study reveals a novel mechanistic role for HOXA7 in modulating granulosa cell proliferation via the regulation of EGFR. This finding contributes to the knowledge of the pro-proliferation effect of HOXA7 in granulosa cell growth and differentiation.

## Background

Ovarian follicular maturation represents one of the most complex and clinically important developmental processes during the reproductive life of women. Granulosa cells surround the developing oocyte, providing a critical microenvironment for follicular growth. Multiple granulosa cell dysfunctions lead to disordered ovulatory and ovarian function [1]. Moreover, granulosa cell tumors (GCTs) are serious ovarian neoplasms that can occur in women of all ages [2]. As most malignant ovarian tumors are epithelial in origin, most studies of ovarian cancer do not include GCTs [3]. Furthermore, while much is now known about the biology of normal granulosa cells [4], the molecular changes that contribute to human granulosa cell dysfunction remain to be elucidated.

Homeobox (HOX) genes encode evolutionarily conserved transcription factors that are essential for embryonic morphogenesis and differentiation [5]. Mammalians have at least 39 HOX genes that are arranged in four clusters termed HOX A, B, C, and D [6]. HOX genes exert pleiotropic roles in many cell types and can regulate cell proliferation, differentiation, adhesion, and migration [7]. HOX genes play important roles in organogenesis and in the development of the human reproductive system during embryogenesis and during organic remodeling in adults [8]. Recent studies suggest that HOX genes may play important roles in ovarian cancer differentiation [9-11]. However, the role of HOX genes in developing granulosa cells is not well known.

We previously demonstrated that three HOXA genes, HOXA4, HOXA7 and HOXA10, were overexpressed in serous ovarian adenocarcinomas when compared to benign serous tumors or tumors with low malignant potential. Among these genes, HOXA7 was one of the HOX genes most consistently overexpressed in ovarian cancers [12]. Additionally, the expression of HOXA7 was detected in ovarian tumors exhibiting mullerian-like features and correlated with the generation of anti-HOXA7 antibodies in patients [10]. Our studies about the role of HOXA7 in human ovarian folliculogenesis showed that HOXA7 expression was predominantly negative in primordial follicles and positive in primary and mature follicles. Moreover, the subcellular localization of HOXA7 changed from nuclear to predominantly cytoplasmic during follicular maturation [13]. This differential localization indicated that HOXA7 underwent cell type- and stage-specific changes during ovarian folliculogenesis, which likely resulted in the regulation of granulosa cell proliferation. Moreover, the expression of HOX cofactors were also temporally and spatially specific in human granulosa cells, which indicated the specific role of HOXA7 in regulating granulose cell function [14]. However, little is known regarding the specific pathways regulated by HOXA7 that promote the growth and survival of granulosa cells.

Epidermal growth factor receptor (EGFR) belongs to the receptor tyrosine kinase (RTK) family [15]. EGF signaling plays an important role in cell growth and differentiation [16]. A possible function for EGF and EGFR signaling at select stages of follicle maturation has been previously proposed and is supported by many observations of the effects of EGF on steroidogenesis, oocyte maturation, and cumulus expansion [17,18]. The binding of EGF to EGFR leads to receptor dimerization, autophosphorylation and the activation of several downstream signaling pathways, such as the MAPK pathway and the PI3K/Akt pathway, which play roles in cell proliferation, motility, and survival [19]; these pathways have also been shown to contribute to the abnormal growth of several types of human cancers [20]. Recent reports have demonstrated that HOX genes play a role in the regulation of several RTK family members, including EGFR [21], IGF1-receptor [22], and Eph-receptor [23,24], during development. Moreover, EGFR activation has been reported to stimulate HOXA7 expression [25].

In this study, we used siRNA and overexpression approaches to define the role of HOXA7 in the regulation of granulosa cell proliferation. Primary granulosa cells (hGCs), an immortalized human granulosa cell line, SVOG, and a granulosa tumor cell line, KGN, were used as cell models. The KGN cell line (stocked in the RIKEN CELL Bank) was derived from a human ovarian granulosa cell tumor, which expresses the functional FSH receptor and maintains the functions of steroidogenesis and Fas-mediated apoptosis [26]. The SVOG cell line was produced by transfecting granulosa cells with SV40 Tag as described previously [27]. We show that the down-regulation of HOXA7 results in decreased EGFR expression and cell proliferation. Conversely, EGFR expression and cell proliferation were increased by the overexpression of HOXA7. Our findings suggest that HOXA7 is crucial for granulosa cell proliferation and may exert its function by regulating the expression of EGFR. These results are the first to functionally demonstrate a link between HOXA7 expression, the regulation of proliferation and EGFR expression in human granulosa cells.

## Methods

### Cells and cell culture

Primary human granulosa cells (hGCs) were obtained with the approval of the Research Ethics Board of the University of British Columbia. hGC isolation from follicular aspirates collected during oocyte retrieval from women undergoing *in vitro *fertilization was performed as previously described [28]. In each well of six-well culture plates, 3 × 10^5 ^viable cells were seeded and cultured in a humidified atmosphere of 5% CO_2_-95% air at 37°C in 1:1 Dulbecco modified Eagle medium and Ham's F-12 medium (DMEM/F12; Sigma, St. Louis, MO, USA) supplemented with 10% fetal bovine serum (FBS; Hyclone Laboratories, Logan, UT, USA), 100 U/mL penicillin G and 0.1 mg/mL streptomycin sulfate (Invitrogen, Burlington, ON, CA). After 48 h, the hGCs were harvested from the plate for RNA or protein extraction.

KGN cells were maintained in 1:1 DMEM/F12, supplemented with 10% FBS, 100 U/mL penicillin, and 0.1 mg/mL streptomycin. The SVOG cells were maintained in M199/MCDB105 (Invitrogen) supplemented with 10% FBS, 100 U/mL penicillin G, and 0.1 mg/mL streptomycin. In each well of six-well culture plates, 2 × 10^5 ^viable KGN or SVOG cells were seeded, and cultures were maintained at 37°C in a humidified atmosphere of 5% CO_2_. The cells were sub-cultured when they reached about 90% confluence using a trypsin/EDTA solution (0.05% trypsin, 0.5 mM EDTA).

### Antibodies and reagents

The monoclonal HOXA7 antibody, polyclonal EGFR antibody and polyclonal β-actin antibody were obtained from Santa Cruz Biotechnology (Santa Cruz, CA, USA). The monoclonal phospho-ERK1/2 (Thr202/Tyr204) antibody, polyclonal total ERK1/2 antibody, polyclonal phospho-Akt (Ser473) and polyclonal total-Akt antibody were obtained from Cell Signaling Technology (Danvers, MA, USA). Horseradish peroxidase-conjugated goat anti-mouse IgG and goat anti-rabbit IgG were obtained from Bio-Rad Laboratories (Hercules, CA, USA). Horseradish peroxidase-conjugated donkey anti-goat IgG was obtained from Santa Cruz. Human epidermal growth factor (EGF) and the EGFR inhibitor, AG1478, were obtained from Sigma.

### Small interfering RNA (siRNA) transfection

KGN cells were cultured in growth medium as described above to achieve approximately 50-60% confluence at the time of transfection. Transfections were performed using a non-targeting negative control siRNA (Dharmacon, Chicago, IL, USA) or siRNAs against human HOXA7 (ON-TARGET plus SMART pool, Dharmacon) at a final concentration of 75 nM using Lipofectamine RNAiMAX (Invitrogen). Following transfection, the cells were directly processed for mRNA/protein extraction at different time points (24 h, 48 h and 72 h after transfection), or reseeded into new culture plates after 36 h of transfection for further cell viability analysis (24 h, 48 h, 72 h and 96 h after reseeding) or EGF treatment for various periods of time (48 h for cell viability analysis and 10 min for signaling pathway detection).

### Plasmid constructs and transfection

Full-length HOXA7 (917 bp) was amplified by RT-PCR using the Phusion RT-PCR Kit (New England BioLabs, Ipswich, MA, USA) and the following primers (5'-3'): TCA TTC CTC CTC GTC C and ATG AGT TCT TCG TAT TAT GAA CG. Gel-purified PCR product was cloned into the pcDNA3.1 (+) vector using the pCR- Blunt II-TOPO Expression Kit (Invitrogen) and confirmed by DNA sequencing (Child & Family Research Institute DNA Sequencing Core Facility, Vancouver, BC, CA). pcDNA3.1/CAT was used as a control vector. SVOG cells were cultured in growth medium as described above to achieve approximately 80-90% confluence at the time of transfection. Transient transfections were carried out using Lipofectamine 2000 Reagent (Invitrogen) following the manufacturer's protocol. After transfection, cells were directly processed for mRNA/protein extraction at different time points, or reseeded into new culture plates after 36 h of transfection for further cell viability analysis or EGF treatment for various periods of time, as described above.

### Cell growth and proliferation

Cell growth was evaluated via the 3-(4, 5-dimethyl thiazol-2-yl)- 2,5-diphenyl tetrazolium bromide (MTT) assay. Briefly, 1 × 10^4 ^cells were seeded in triplicate in 96-well plates. In some experiments, cells with or without HOXA7 transfection were treated with EGF, AG1478, or EGF and AG1478. At the indicated times, a final concentration of MTT at 500 μg/mL (Sigma) was added and the cells were incubated for an additional 4 h at 37°C. Following the incubation period, DMSO was added to dissolve the crystals. The absorbance was measured at 492 nm and was expressed as the relative proliferation rate.

### RNA extraction and Real-time PCR

At the end of the treatment period, the medium was removed from the culture plate and RNA was extracted using TRIzol (Invitrogen). RNA concentration was measured based on the absorbance at 260 nm. The isolated RNA was reverse transcribed into first-strand cDNA using M-MLV reverse transcriptase (Promega BioSciences, San Luis Obispo, CA, USA). The primers used for SYBR Green real-time RT-PCR were designed using Primer Express Software v2.0 (PerkinElmer Applied Biosystems, Foster City, CA, USA). The primers for HOXA7 are: sense, 5'-TAC CCC TGG ATG CGG TCT T-3'; and antisense, 5'-CAG GTA GCG GTT GAA GTG GAA-3'. The primers for EGFR are: sense, 5'-CAT GTC GAT GGA CTT CCA GA-3'; and antisense, 5'-GGG ACA GCT TGG ATC ACA CT-3'. The primers for GAPDH are: sense, 5'-ATG GAA ATC CCA TCA CCA TCT T-3'; and antisense, 5'-CGC CCC ACT TGA TTT TGG-3'. Real-time PCR was performed on the ABI PRISM^® ^7300 Sequence Detection System according to the manufacturer's protocol (Applied Biosystems). Amplification specificity was determined using the melting curve. Analysis and quantification of the relative mRNA levels was detected using the comparative Ct method. All real-time experiments were run in triplicate, and a mean value was used for the determination of mRNA levels. Negative controls containing water were used in each experiment. Relative quantification of the mRNA levels for HOXA7 and EGFR in granulosa cells was performed using the comparative Ct method with GAPDH as an endogenous control. Changes following treatments were recorded as fold differences from the values in untreated controls at each time point, as appropriate, with the formula 2^-ΔΔCt^.

### Western blot analysis

After treatment, cells were washed twice with ice-cold PBS and harvested in ice-cold lysis buffer (20 mM Tris-HCl, 150 mM NaCl, 1 mM EDTA, 1 mM EGTA, 1% Triton, 2.5 mM sodium pyrophosphate, 1 mM beta-glycerophosphate, 1 mM Na_3_VO_4_, 1 μg/mL leupeptin) containing protease inhibitors (Sigma). The extract was centrifuged at 14,000 rpm for 15 min at 4°C to remove cellular debris. Protein concentrations were determined by the Bradford method (Bio-Rad Laboratories). SDS-PAGE was used to further separate 30 μg of protein sample, which was then transferred onto nitrocellulose membranes (Bio-Rad Laboratories). The membranes were blocked for 1 h in Tris-buffered saline containing 0.01% Tween 20 with 5% non-fat dried milk and were incubated overnight at 4°C with the relevant primary antibodies. After washing, the membranes were incubated with the secondary antibody, a peroxidase-conjugated anti-IgG for 1 h. Immunoreactive proteins were detected using enhanced chemiluminescence reagents (Amersham Bioscience, Baied'Urfe, Quebec, CA). For the detection of antibody bound to HOXA7, we used the SuperSignal Femto West (Pierce Chemical. Rockford, IL, USA) reagent as described by the manufacturer. β-actin was used as the internal control.

### Statistical analysis

Data are presented as the means ± SEM from at least three independent experiments. Data were analyzed by one-way ANOVA, followed by Tukey's multiple comparison tests if the overall P values were significant, using the computer software PRISM (GraphPad Software, San Diego, CA, USA). Data were considered significantly different from each other at P < 0.05.

## Results

### HOXA7 and EGFR are expressed in human granulosa cells

**Figure 1:**
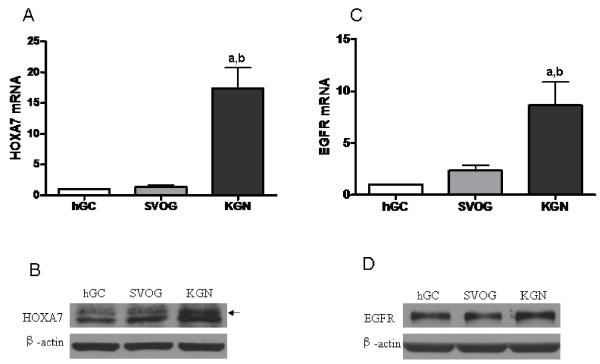
**HOXA7 and EGFR expression in human granulosa cells**. Expression of HOXA7 and EGFR was detected in primary hGCs, the immortalized human granulosa cell line, SVOG, and the granulosa tumor cell line, KGN, cells by both real-time PCR and Western blotting. (A) Real-time PCR results of relative HOXA7 mRNA expression. (B) The representative autoradiographs of HOXA7 detected by Western blotting (indicated by arrow). (C) Real-time PCR results of relative EGFR mRNA expression. (D) The representative autoradiographs of EGFR detected by Western blotting. The data derived from at least three separate sets of experiments were standardized to the corresponding control, and the statistical results are presented in the column graphs. a, *P *< 0.05 compared with hGC; b, *P *< 0.05 compared with SVOG.

Expression of HOXA7 was detected in hGCs, SVOG and KGN cells by both real-time PCR and Western blotting. The expression level of HOXA7 in the granulosa tumor cell line KGN was significantly higher than in primary granulosa cells and the immortalized human granulosa cell line, SVOG (Fig. 1, A and 1B). EGFR expression was also detected in all cell lines. Consistent with HOXA7, KGN cells expressed significantly higher levels of EGFR compared to hGCs and SVOG cells (Fig. 1, C and 1D).

### HOXA7 regulates granulosa cell proliferation

**Figure 2:**
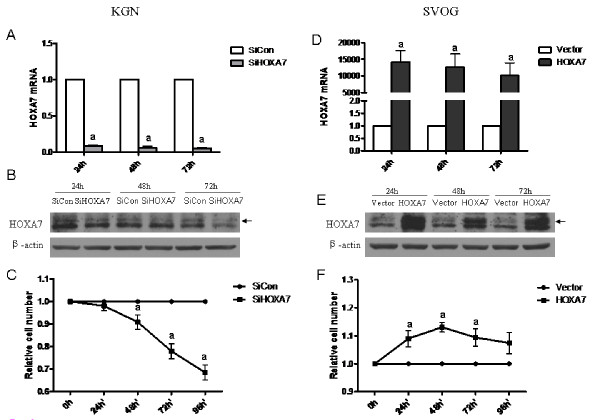
**HOXA7 regulates granulosa cell proliferation**. KGN cells were transiently transfected with scrambled siRNA or HOXA7 siRNA and the effect of HOXA7 knockdown was verified at the mRNA level by real-time PCR (A) and protein level by Western blotting (B). (C) After 36 h of transfection, KGN cells were reseeded into a 96-well plate and the cell viability was detected by MTT assay. SVOG cells were transiently transfected with control vector or HOXA7 plasmid and the effect of HOXA7 overexpression was verified at the mRNA level by real-time PCR (D) and protein level by Western blotting (E). (F) After 36 h of transfection, SVOG cells were reseeded into a 96-well plate and the cell viability was detected by MTT assay. The data derived from at least three separate sets of experiments were standardized to the corresponding control. a, *P *< 0.05 compared with control siRNA or control vector.

To determine the functional role of endogenous HOXA7, we used siRNA to knockdown the expression of HOXA7 in KGN cells, which expressed high levels of HOXA7. HOXA7 knockdown was verified at the mRNA level by real-time PCR (Fig. 2, A) and at the protein level by Western blotting (Fig. 2, B). To examine the role of HOXA7 in granulosa cell proliferation, KGN cells transfected with HOXA7 siRNA or control siRNA were processed for the MTT assay. In response to HOXA7 knockdown, the proliferation rate of KGN cells was significantly decreased (Fig. 2, C).

To further determine the role of HOXA7 in human granulose cells, overexpression of HOXA7 was performed in the SVOG cell line, which expressed low levels of HOXA7. HOXA7 overexpression in SVOG cells was also verified at both the mRNA and protein levels by real-time PCR and Western blotting, respectively (Fig. 2, D and 2E). Overexpression of HOXA7 significantly increased cell proliferation in SVOG cells (Fig. 2, F).

### HOXA7 regulates EGFR expression in granulosa cells

**Figure 3:**
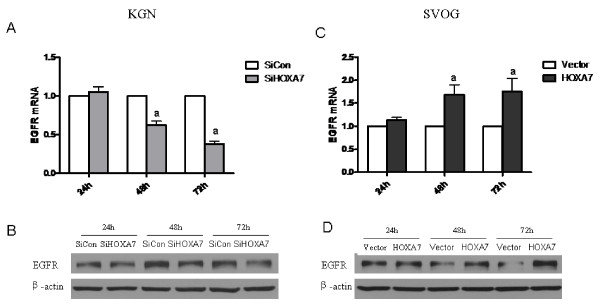
**HOXA7 regulates EGFR expression in granulosa cells**. KGN cells were transiently transfected with scrambled siRNA or HOXA7 siRNA. Expression of EGFR was detected at the mRNA level by real-time PCR (A) and protein level by Western blotting (B). SVOG cells were transiently transfected with control vector or HOXA7 plasmid and expression of EGFR at the mRNA level was detected by real-time PCR (C) and at the protein level by Western blotting (D). The data derived from at least three separate sets of experiments were standardized to the corresponding control, and the statistical results are presented in the column graphs. a, *P *< 0.05 compared with control siRNA or control vector.

The expression level of EGFR in granulosa cells after HOXA7 knockdown or overexpression was detected at both the mRNA level and protein level. Knockdown of endogenous HOXA7 in KGN cells significantly reduced EGFR expression, especially after 48 h of transfection (Fig. 3, A and 3B). Conversely, HOXA7 overexpression in SVOG cells significantly increased EGFR mRNA and protein levels, especially after 48 h of transfection (Fig. 3, C and 3D).

### EGF induces granulosa cell proliferation via EGFR-mediated MAPK and PI3K/Akt activation

**Figure 4:**
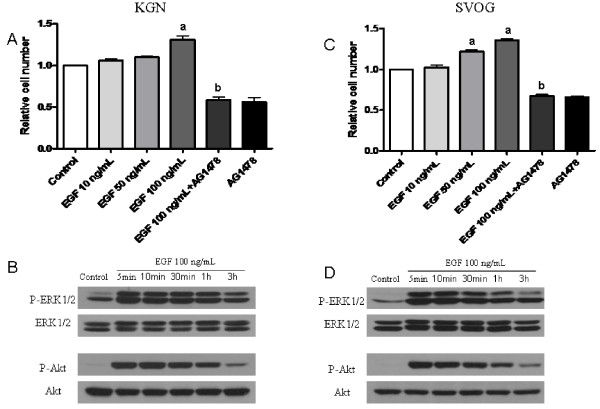
**EGF induces granulosa cell proliferation via the activation of the MAPK and PI3K/Akt pathways**. KGN and SVOG cells were serum starved for 12 h and incubated in serum-free medium containing various concentrations of EGF and/or AG1478 for various periods of time. KGN cells (A) and SVOG cells (C) were left untreated (Control) or treated with increasing concentrations of EGF and/or AG1478 for 48 h. The viability was measured by the MTT assay. The data derived from at least three separate sets of experiments were standardized to the corresponding control, and the statistical results are presented in the column graphs. a, *P *< 0.05 compared with control; b, *P *< 0.05 compared with treatment with EGF 100 ng/mL alone. KGN cells (B) and SVOG cells (D) were treated with 100 ng/mL EGF for 5 min, 10 min, 30 min, 1 h, 3 h or were untreated (Control). The phosphorylation of ERK1/2 or Akt was determined by Western blotting with specific antibodies.

Cell viability assays showed that EGF promoted KGN and SVOG cell proliferation, especially at the concentration of 100 ng/mL (Fig. 4, A and 4C). Therefore, 100 ng/mL EGF was used in the subsequent experiments. The stimulatory effects of EGF could be abolished by the co-treatment with 1 μM of the specific EGFR inhibitor, AG1478 (Fig. 4, A and 4C). In addition, Western blotting results demonstrated that the treatment of both KGN and SVOG cells with EGF induced the phosphorylation of ERK1/2 and Akt, suggesting that the MAPK and PI3K/Akt signaling pathways play a role in mediating the induction of proliferation by EGF in the granulosa cell lines (Fig. 4, B and 4D).

### EGF-induced granulosa cell proliferation and downstream signaling activation could be modulated by the expression of HOXA7

**Figure 5:**
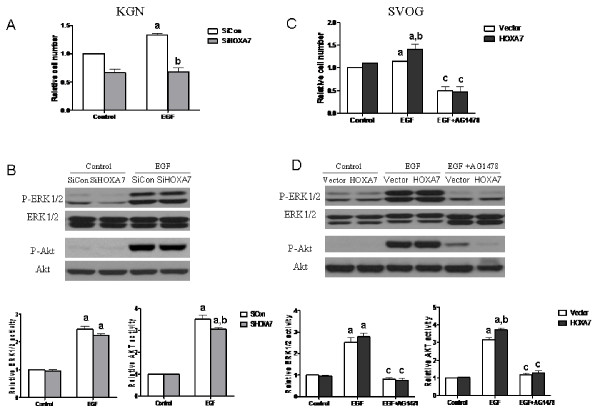
**EGF-induced granulosa cell proliferation and activation of downstream signaling are modulated by the expression of HOXA7**. KGN cells were transiently transfected with scrambled siRNA or HOXA7 siRNA. SVOG cells were transiently transfected with control vector or HOXA7 plasmid. KGN cells (A) and SVOG cells (C) were reseeded into a 96-well plate after 36 h of transfection. Cells were serum starved for 12 h and incubated in serum-free medium containing 100 ng/mL EGF and/or AG1478, or were left untreated (Control) for 48 h. Cell viability was measured by the MTT assay. KGN cells (B) and SVOG cells (D) were serum starved for 12 h after 36 h transfection and incubated in serum free medium containing 100 ng/mL EGF and/or AG1478, or left untreated (Control) for 10 min. The phosphorylation of ERK1/2 or Akt was determined by Western blotting with specific antibodies. The data derived from at least three separate sets of experiments were standardized to the corresponding control, and the statistical results are presented in the column graphs. a, *P *< 0.05 compared with control; b, *P *< 0.05 compared with control siRNA or control vector transfection and EGF treatment. c, *P *< 0.05 compared with EGF treatment alone.

Treatment with EGF significantly induced proliferation of KGN cells; however, this effect was abrogated when HOXA7 was knocked down by siRNA transfection (Fig. 5, A). In addition, the EGF-induced phosphorylation of Akt was diminished after HOXA7 knockdown. Quantitative analysis showed that, while the basal phosphorylation was not changed, the EGF-induced phosphorylation of Akt was significantly decreased (Fig. 5, B). In contrast, EGF-induced cell proliferation was enhanced in SVOG cells after HOXA7 overexpression, and this effect could be completely abolished by co-treatment with AG1478 (Fig. 5, C). Accordingly, the EGF-induced phosphorylation of Akt was significantly increased after HOXA7 overexpression, and this effect could also be abolished by treatment with AG1478. However, the knockdown or overexpression of HOXA7 did not significantly change the phosphorylation of ERK1/2 in either cell line (Fig. 5, B and 5D).

## Discussion

HOX genes are regulatory genes encoding nuclear proteins that act as transcription factors, which regulate aspects of morphogenesis and cell differentiation. Spatiotemporal aberrations in HOX gene expression have been found with polycystic ovarian syndrome (PCOS), endometriosis, hydrosalpinges, and endocrine disrupters that compromise reproduction [8,29]. Moreover, there is accumulating evidence that HOX genes play important roles in oncogenesis, including ovarian cancer progression [30]. However, the specific role of HOX genes in granulosa cell function and oncogenesis has not been extensively explored. In this study, we show for the first time a significant role for HOXA7 in the regulation of granulosa cell proliferation and EGFR expression. These findings indicate a new mechanism for HOX-mediated cell proliferation that may act through the regulation of EGFR expression.

We initially analyzed the expression level of HOXA7 in human granulosa cells. We first demonstrated that all three kinds of granulosa cells express HOXA7. Unlike most HOX genes, which are expressed solely during the embryonic period, those specific to the female reproductive tract continue to play a role in the adult [8,31]. The expression of HOXA7 in granulosa cells indicated that HOXA7 exerted a potential role in cell regulation. In the current study, KGN cells were used as a granulosa tumor cell model and SVOG cells were used as a normal granulosa cell model. Although SVOG cells were derived from granulosa-luteal cells, it is still a good model for the study of human granulosa cells as it is difficult to get human granulosa cells at other stages [27]. Quantitative analysis showed the expression level of HOXA7 was significantly higher in the granulosa tumor cell line, KGN, compared to primary hGCs and SVOG cells. It has been reported that many cancers, including leukemia, colon, skin, prostate, breast and ovarian cancers, have shown alterations in the expression patterns of HOX genes [30,32]. The overexpression of HOX genes has been widely associated with a variety of ovarian carcinomas. Therefore, our results are consistent with previous observations that HOX genes are highly expressed in a subset of ovarian cancers. HOXA7, in particular, plays a role in granulosa cell growth and differentiation.

In the present study, we performed siRNA-mediated knockdown of HOXA7 in the high HOXA7-expressing KGN cell line. Conversely, we overexpressed HOXA7 in the low HOXA7-expressing SVOG cell line to determine how HOXA7 regulates granulosa cell growth. The results show that HOXA7 exerted a specific role in granulosa cell proliferation: knockdown of HOXA7 induced a decrease in KGN proliferation, while overexpression of HOXA7 in SVOG cells significantly promoted cell growth. A previous study indicated that HOXA7 expression was increased during mitosis in cultured granulosa cells. Moreover, growth differentiation factor-9 (GDF-9), which enhances early follicular growth and differentiation, increased HOXA7 protein expression and regulated the expression of HOXA7 cofactors in granulosa cells [13,14]. Overexpression of HOXA7 was also detected in ovarian carcinomas [12]. These results support the hypothesis that HOXA7 modulates the growth and oncogenesis of human granulosa cells.

On the other hand, EGFR was also found to be overexpressed in granulosa tumor cells. EGFR is a transmembrane receptor tyrosine kinase known to be involved in numerous cellular processes such as growth, motility and cellular proliferation. EGF stimulates the replication of granulosa cells, thereby inducing follicle growth [33]. The expression levels of EGFR have been correlated with the pathogenesis of a broad range of human cancers [34]. EGFR overexpression has been found to be a marker of poor prognosis [35,36]. In the current study, we examined the effect of EGF on granulosa cell growth. As observed by others in multiple cell types [37,38], EGFR activation in granulosa cells promoted cell proliferation and stimulated the classical ERK1/2 and PI3K/Akt pathways. Those effects could be blocked by the specific EGFR inhibitor, AG1478. Our study further identified EGFR as a novel downstream target of HOXA7.

While HOXA7 regulation of EGFR expression has not been extensively studied, several reports have previously demonstrated a relationship between receptor tyrosine kinases and HOX genes. Henderson *et al*. reported that HOXA5 protein expression levels in breast carcinomas inversely correlated with EGFR expression [21]. Bruhl *et al*. reported that HOXA9 bound to the EphB4 promoter and stimulated its expression, resulting in an increase in endothelial cell migration [23]. Recently, Whelan *et al. *indicated that HOXA9 overexpression induced the expression of another RTK member, the IGF-1 receptor, and subsequently promoted leukemic cell growth [22]. In the present study, we showed that the down-regulation of HOXA7 significantly reduced EGFR mRNA and protein expression in KGN cells. In contrast, overexpression of HOXA7 significantly increased EGFR mRNA and protein expression in SVOG cells. Furthermore, our results indicated that EGF-induced granulosa cell proliferation and downstream signaling activation, especially in the PI3K/Akt pathway, could be modulated by the expression of HOXA7. These data suggest that HOXA7 regulates the expression of the EGF receptor in granulosa cells and, consequently, modulates the cell proliferative capacity. However, the detailed molecular mechanism of HOXA7-regulated EGFR expression remains to be further elucidated.

EGFR deregulation has been shown to be associated with aberrant oocyte maturation and cumulus expansion [39]. EGFR has also been shown to be highly expressed in numerous types of human cancers. Therefore, the level of EGFR expression can act as a strong prognostic factor [35,36]. EGF is a critical regulator of cell survival responses and downstream signaling events leading to cell survival, thus making it an attractive target for disease therapy [37,40]. Several monoclonal antibodies or small molecule inhibitors directed against EGFR have been successfully used in cancer therapy [41,42]. Our findings herein suggest that the inhibition of EGFR, either as a stand-alone therapy or in combination with other approaches, might be a good approach for the treatment of granulosa cell proliferation dysfunction, granulosa cell tumors or other cancers that exhibit elevated HOX gene expression. Certainly, EGF signaling alone is not sufficient to maintain cell survival indefinitely. HOXA7 may also affect additional proteins. Therefore, our observations and the interest in granulosa cell therapies require further investigation at the clinical and basic science levels.

In summary, our present data support a novel mechanistic role for the HOXA7 modulation of granulosa cell proliferation via the expression of EGFR. This finding contributes to the pro-proliferation effect of HOXA7 in granulosa cell growth and differentiation. This pathway will yield new potential targets for the treatment of granulosa cell tumors and other granulosa cell proliferation dysfunction that involve the aberrant expression of HOX genes.

## Competing interests

The authors declare that they have no competing interests.

## Authors' contributions

YZ and QH participated together with HFH and PCKL in the design of the study. YZ and JCC carried out the experiments. Data analysis was performed by YZ and QH. The manuscript was written by YZ. YN, TY, HFH and PCKL critically read the manuscript. All authors read and approved the final manuscript.
